# The Hypoxia Tolerance of the Goldfish (*Carassius auratus*) Heart: The NOS/NO System and Beyond

**DOI:** 10.3390/antiox9060555

**Published:** 2020-06-26

**Authors:** Mariacristina Filice, Rosa Mazza, Serena Leo, Alfonsina Gattuso, Maria Carmela Cerra, Sandra Imbrogno

**Affiliations:** Department of Biology, Ecology and Earth Sciences, University of Calabria, 87036 Arcavacata di Rende (CS), Italy; mariacristina.filice@unical.it (M.F.); rosa.mazza@unical.it (R.M.); serena.leo@unical.it (S.L.); alfonsina.gattuso@unical.it (A.G.)

**Keywords:** myocardial performance, cGMP, SERCA2a, Nox2, nitrosative signals

## Abstract

The extraordinary capacity of the goldfish (*Carassius auratus*) to increase its cardiac performance under acute hypoxia is crucial in ensuring adequate oxygen supply to tissues and organs. However, the underlying physiological mechanisms are not yet completely elucidated. By employing an ex vivo working heart preparation, we observed that the time-dependent enhancement of contractility, distinctive of the hypoxic goldfish heart, is abolished by the Nitric Oxide Synthase (NOS) antagonist L-NMMA, the Nitric Oxide (NO) scavenger PTIO, as well as by the PI3-kinase (PI3-K) and sarco/endoplasmic reticulum Ca^2+^-ATPase 2a (SERCA2a) pumps’ inhibition by Wortmannin and Thapsigargin, respectively. In goldfish hearts exposed to hypoxia, an ELISA test revealed no changes in cGMP levels, while Western Blotting analysis showed an enhanced expression of the phosphorylated protein kinase B (pAkt) and of the NADPH oxidase catalytic subunit Nox2 (gp91phox). A significant decrease of protein S-nitrosylation was observed by Biotin Switch assay in hypoxic hearts. Results suggest a role for a PI3-K/Akt-mediated activation of the NOS-dependent NO production, and SERCA2a pumps in the mechanisms conferring benefits to the goldfish heart under hypoxia. They also propose protein denitrosylation, and the possibility of nitration, as parallel intracellular events.

## 1. Introduction

Hypoxia is a stress condition threatening to life. In aquatic systems, it results from complex converging processes which include mixing, air–water exchange, respiration, and variations in the amount of O_2_ production and consumption [1,2]. In many cases, these processes are altered by anthropogenic and climate changes, thus leading to prolonged low-oxygen conditions, with severe consequences on aquatic organisms [3,4].

Several fish species have evolved the ability to inhabit hypoxic, and even anoxic, environments. Major adaptations include metabolic depression, acidosis tolerance, and reoxygenation injury prevention (see for review [5,6]).

Members of the cyprinid genus *Carassius* [i.e., the crucian carp (*Carassius carassius*) and the goldfish (*Carassius auratus*)] are able to survive and remain active for long periods under hypoxia, even tolerating a complete lack of O_2_ [5]. Thus, they represent valuable experimental models for studying the physiological strategies that allow animals to survive with reduced oxygen. In several fish species, the resistance to protracted hypoxia/anoxia is supported by a preserved cardiac activity and autonomic cardiovascular control [7,8,9]. This allows them not only to mobilize glucose from hepatic glycogen stores to all tissues, but also to transport lactate to the muscle, where it is converted into the less harmful ethanol, rapidly removed via the branchial epithelium [10,11]. In this context, our previous studies have documented that under acute hypoxia, the goldfish improves its cardiac performance [12], this representing a crucial physiological compensatory mechanism to support its hypoxia tolerance [10]. However, the specific molecular pathways involved remain elusive.

The Nitric Oxide Synthase (NOS)/Nitric Oxide (NO) system is a pleiotropic cardiac regulator. NOS isoenzymes [i.e., constitutive endothelial (e)NOS and neuronal (n)NOS, and inducible (i)NOS], convert l-arginine into l-citrulline and NO, by using molecular O_2_ and NADPH as cofactors. Constitutive NOSs generate nanomolar concentrations of NO, while iNOS produces micromolar cytotoxic amounts of the gas. NO is rapidly metabolized to nitrite and nitrate which, under reduced O_2_, can be reconverted to NO [13,14], thus contributing to NO homeostasis.

NO exerts its physiological effects by reacting with hemes, thiols or amines, to produce iron-nitrosyl (FeNO), S-nitroso (SNO) and N-nitroso (NNO) compounds [15]. It can also react with the superoxide anion to produce peroxynitrite (ONOO^-^) [16], a highly reactive product [17] able to form additional reactive nitrogen species (RNS). RNS may induce protein post-translational modifications through either S-nitrosation [i.e., the reaction between a NO^+^ equivalent and a nucleophilic center (amine or thiol)], or S-nitrosylation [i.e., the addition of NO to a reactant without changing the formal charge of the substrate (metal centers or radical species)] [18]. Under hypoxia, NO may also determine protein nitration. This consists in the substitution, mainly under the action of ONOO^-^, of a nitro group to tyrosine residues, to give 3-nitrotyrosine (3-NT) [19]. Uncontrolled nitrosation/nitrosylation/nitration may cause nitrosative stress, with important consequences for protein activity, stability, conformation and/or interaction with other molecules [20].

The influence elicited by NO on the heart has been widely assessed in mammals, and also in fish, for which many data are now available (see for example [21,22,23,24,25,26,27,28,29,30]). More recently, the role of NO in fish has been extended to the mechanisms which maintain cardiac health under hypoxia (see, for references, [10]). It has been revealed that, when the NOS activity is compromised by low O_2_, an increased NOS expression and/or a nitrite/nitrate conversion to NO, will stabilize NO levels, and this is protective for the hypoxic myocardium [12,13,31,32]. However, so far, limited pieces of evidence are available concerning the intracellular signals activated by NO during hypoxia.

Moved by these premises, and by taking advantage of the goldfish *C. auratus* as a natural model of hypoxia resistance, the present study was designed to furnish a deeper insight into the NO targets and downstream events activated in cardiac cells under low O_2_. The goldfish heart, although possessing a very thin outer compact layer with coronaries, is primarily composed of spongy myocardium, mainly dependent on intracavitary blood perfusion for oxygen supply [33]. This trait, as in other teleost hearts (see [34] and references therein), makes it particularly suited for this type of study [35].

By using an ex vivo working goldfish heart preparation, we showed that the hypoxia-dependent increase of cardiac contractility, distinctive of this cyprinid, requires a PI3-K/Akt/NOS-dependent NO production, and the activation of SERCA2a pumps, while it is independent of cGMP-mediated transduction pathways. We also found that hypoxia exposure is accompanied by a protein S-denitrosylation and an increased expression of the Nox2 (gp91phox) catalytic subunit of the NADPH oxidase.

## 2. Materials and Methods

### 2.1. Animals

Goldfish (*C. auratus*; length = 12–16 cm; weight = 47.15 ± 3.72 g; means ± s.e.m.) specimens of both sexes were provided by local hatcheries. Fish were maintained at 18–21 °C in filtered and aerated water, 12 h light/dark cycle, and daily fed with commercial food. Before the sacrifice, they were anesthetized with MS222 (tricaine methanesulfonate; 0.2 g L^−1^) (Sigma–Aldrich, Milan, Italy Animal care and experimental procedures were in accordance with the U.S. National Institutes of Health’s Guide for the Care and Use of Laboratory Animals (NIH Publication No. 85–23, revised 1996), with the European Directive (2010/63/EU) and with the Italian law (DL 116, January 27, 1992), which did not require a specific authorization for the used species by an ethics committee. 

### 2.2. Isolated and Perfused In Vitro Working Heart Preparations

Hearts were isolated, cannulated and connected to a perfusion apparatus as described [12,36]. They were perfused with a solution containing (in mmol L^−1^) NaCl 124.9, KCl 2.49, MgSO_4_ 0.94, NaH_2_PO_4_ 1.0, Glucose 5.0, NaHCO_3_ 15.0, and CaCl_2_ 1.2, equilibrated with a mixture of either 99.5% O_2_ and 0.5% CO_2_ (normoxia), or 10% O_2_, 0.5% CO_2_ and 89.5% N_2_ (hypoxia) [8]. The pH was adjusted to 7.7–7.9. Experiments were performed at room temperature (18–20 °C). Oxygen concentration in the input reservoir was continuously monitored by an oxygen analyzer (Milwaukee, SM600, Szeged, Hungary). Concentrations of 8.4 ± 0.2 mg O_2_ L^−1^ (normoxia), and 2.5 ± 0.3 mg O_2_ L^−1^ (hypoxia), means ± s.e.m., were chosen on the basis of previous data [8,37].

Hearts were electrically paced with a LE 12006 stimulator (frequency identical to control, non-paced hearts; pulse width fixed at 0.1 ms; voltage: 1.2 ± 0.1 V; means ± s.e.m.). Pressures were measured with a MP-20D pressure transducer (Micron Instruments, Simi Valley, CA, USA), connected to a PowerLab data acquisition system, and analyzed using Chart software (ADInstruments Basile, Comerio, Italy). Pressure values were corrected for cannula resistance. Cardiac Output (CO) was collected over 1 min and weighed. Values were corrected for fluid density and expressed as volume measurements normalized for body weight (mL min^−1^ kg^−1^). Heart rate (HR, beat min^−1^) was obtained from pressure traces. Stroke Volume (SV; mL kg^−1^; CO/HR) and Stroke Work [SW; mJ g^−1^; (afterload–preload) SV/ventricle mass] were used as indexes of ventricular performance and systolic function, respectively.

### 2.3. Experimental Protocols

#### 2.3.1. Basal Conditions

The isolated and perfused goldfish heart was allowed to maintain a spontaneous rhythm for up to 15–20 min. For control conditions, afterload was set to 1.5 kPa, and CO to 10–14 mL min^−1^ kg^−1^ body mass, by appropriately adjusting output and filling pressure, respectively [33]. Cardiac variables were simultaneously measured during experiments. Hearts that did not stabilize within 20 min of perfusion were discarded. For time-course experiments, cardiac parameters were measured every 10 min with either normoxic or hypoxic perfusion medium, for about 90 min of perfusion.

#### 2.3.2. Drug Application

After stabilization, ex vivo cardiac preparations were perfused in the presence of either the NO scavenger 2-phenyl-4,4,5,5-tetramethylimidazolineoxyl-1-oxyl-3-oxide (PTIO; 10^−6^ M), or the NOS enzymes inhibitor NG monomethyl-l-arginine (L-NMMA; 10^−5^ M), or the PI3-kinase (PI3-K) antagonist Wortmannin (10^−9^ M), or the SERCA2a pumps inhibitor Thapsigargin (10^−7^ M). Based on preliminary dose-response curves, inhibitor concentration was the highest dose that did not significantly affect the goldfish basal cardiac performance.

#### 2.3.3. Drugs and Chemicals

L-NMMA was purchased from Sigma–Aldrich. Thapsigargin, Wortmannin and PTIO were from Calbiochem (VWR International, Milan, Italy). L-NMMA was prepared in double-distilled water. PTIO was dissolved in HEPES buffer (0.4 mg/mL). Thapsigargin and Wortmannin were dissolved in DMSO (maximum final concentration less than 0.1%). At this concentration, DMSO per se did not affect the cardiac performance (data not shown). All dilutions were made in the perfusion solution immediately before use.

### 2.4. Western Blot and Densitometric Analysis

Hearts were homogenized in an ice-cold homogenization buffer (250 mmol L^−1^ sucrose, 30 mmol L^−1^ Tris, 1 mmol L^−1^ EDTA, 1% SDS, pH 7.4), containing a mixture of protease inhibitors (1 mmol L^−1^ aprotinin, 20 mmol L^−1^ phenylmethylsulfonyl fluoride and 200 mmol L^−1^ sodium ortho-vanadate). Homogenates were centrifuged at 10,000× *g* for 10 min at 4 °C to remove tissue debris. Protein concentration in the supernatant was determined using Bradford reagent (Sigma–Aldrich) according to the manufacturer. Western Blotting was performed as described [22]. Briefly, a 60-µg protein sample for each homogenate was separated by SDS–PAGE on 10% (w/v) polyacrylamide gels and electroblotted onto a nitrocellulose membrane (GE Healthcare, Milan, Italy). For immunodetection, blot was blocked in TBS-T containing 5% non-fat dry milk and incubated overnight at 4 °C with mouse monoclonal antibody directed against Nox2 (cat# Sc-130543; dilution 1:1000), or rabbit polyclonal antibodies directed against Akt (cat# Sc-8312; dilution 1:500) and pAkt [(Ser473)-pAkt1/2/3 antibody; cat# Sc-7985-R; dilution 1:500)]. Mouse monoclonal Glyceraldehyde-3-Phosphate Dehydrogenase (GAPDH) antibody (cat# Sc-47724; dilution 1:20000) was used as the loading control. All antibodies were from Santa Cruz Biotechnology Inc. (Santa Cruz, CA, USA). Peroxidase linked secondary antibodies (Santa Cruz Biotechnology Inc.) were diluted to 1:1000 in TBS-T containing 5% non-fat dry milk, and incubated for 1h at RT. Immunodetection was performed using an enhanced chemiluminescence kit (ECL PLUS, GE Healthcare). Autoradiographs were scanned to obtain arbitrary densitometric units. Experiments were performed in triplicate; results were expressed as means ± s.e.m. of absolute values.

### 2.5. cGMP Determination

cGMP levels were measured in homogenates from goldfish hearts perfused under both normoxic and hypoxic conditions. Samples were treated with 5% trichloroacetic acid on ice and centrifuged at 1500× *g* for 10 min. The supernatant was extracted three times with 5 volumes of diethyl ether saturated with water; the aqueous phase was collected and used for cGMP measurements, using a commercial enzyme immunoassay kit (cGMP ELISA Kit; Cayman Chemical, Ann Arbor, MI, USA). 

### 2.6. Biotin Switch Assay for Protein s-nitrosylation Assessment

Hearts perfused under normoxic and hypoxic conditions were homogenized on ice in 250 mmol L^−1^ sucrose, 30 mmol L^−1^ Tris, 1 mmol L^−1^ EDTA, 1% SDS, pH 7.4, 200 mmol L^−1^ sodium orthovanadate and Protease Inhibitor Cocktail (Sigma-Aldrich, Milan, Italy). The homogenate was centrifuged at 4 °C for 10 min at 10,000× *g*. The supernatant was collected, and proteins quantified with Bradford reagent. The Biotin Switch assay was performed as in [23]. Samples from the Biotin Switch assay (60 µg of total protein) were separated on 10% (w/v) polyacrylamide gels by SDS–PAGE, transferred to nitrocellulose membrane, blocked with non-fat dried milk, and incubated with streptavidin-peroxidase diluted to 1:1000 for 1 h. For immunodetection, an enhanced chemiluminescence kit (ECL PLUS, GE Healthcare) was used.

### 2.7. Statistics

Physiological data were expressed as means ± s.e.m. of percentage changes obtained from individual experiments. Statistical analysis was performed by using two tailed unpaired *t*-test or one-way ANOVA, followed by Bonferroni’s post-test. Differences were considered statistically significant at *p* < 0.05.

cGMP determination and densitometric analyses were expressed as means ± s.e.m. of absolute values from individual experiments; statistics were assessed by unpaired *t*-test. Significance was concluded at *p* < 0.05.

GraphPad Prism software, version 4.02 (GraphPad Software Inc., San Diego, CA, USA), was used for all statistical analyses.

## 3. Results

### 3.1. Isolated Heart Preparations

The ex vivo isolated and perfused goldfish heart showed basal values of preload, afterload, HR, CO, SV and SW (Table 1) comparable to those previously reported [normoxia: [8,28,36,38]; hypoxia: [8,12,38]].

### 3.2. Role of the NOS/NO System in the Hypoxia-Induced Increase of Contractility

**Figure 1:**
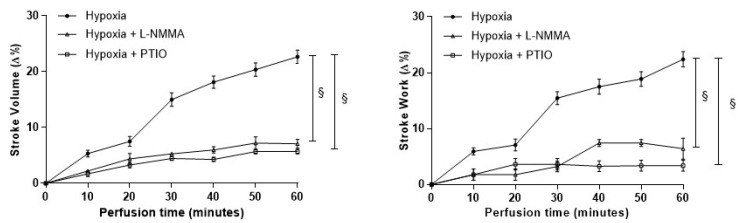
Effects of L-NMMA and PTIO in hypoxia-exposed goldfish hearts. Time-course curves for the Stroke Volume (SV) and Stroke Work (SW) of the isolated and perfused goldfish heart before and after treatment with either L-NMMA (10^−5^ M) or PTIO (10^−6^ M). Data are expressed as mean values ± s.e.m. of 4/7 experiments for each group. Statistics were assessed by one-way ANOVA followed by Bonferroni’s post hoc test (§ *p* < 0.05; hypoxia vs. hypoxia plus either L-NMMA or PTIO).

The perfusion of the goldfish heart under acute hypoxia induces a time-dependent increase of the mechanical performance [12]. To evaluate the involvement of the NOS/NO system, time-course experiments were performed in the presence of the NOS inhibitor L-NMMA (10^−5^ M), or the NO scavenger PTIO (10^−6^ M). Both treatments abolished the hypoxia-dependent increase of contractility (Figure 1), supporting the contribution of a NOS-produced NO.

### 3.3. PI3-K/Akt-Dependent NOS Activation

**Figure 2:**
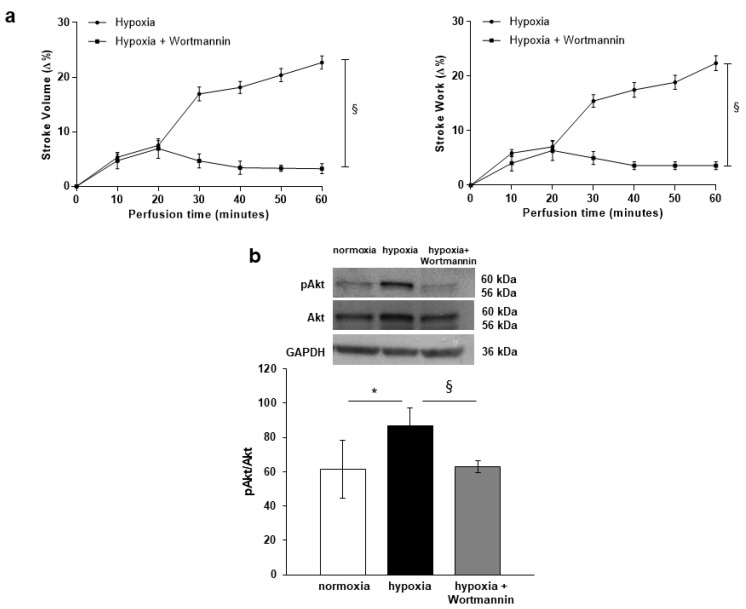
Effects of Wortmannin and pAkt/Akt expression in hypoxia-exposed goldfish hearts. (**a**) Time-course curves for the Stroke Volume (SV) and Stroke Work (SW) of the isolated and perfused goldfish heart before and after treatment with Wortmannin (10^−9^ M). Data are expressed as mean values ± s.e.m. of 4 experiments. Statistics were assessed by two-tailed unpaired *t*-test (§ *p* < 0.05; hypoxia vs. hypoxia plus Wortmannin). (**b**) Representative Western Blotting and densitometric analysis of pAkt (Ser473)/Akt expression in goldfish cardiac extracts under normoxia, hypoxia and hypoxia plus Wortmannin. Data were expressed as means ± s.e.m. of absolute values from individual experiments (*n* = 3); statistics were assessed by two-tailed unpaired *t*-test (* *p* < 0.05).

The PI3-K/Akt pathway plays a relevant role in the NOS activation and the subsequent NO production (for references in fish see [23]). To verify its involvement in the hypoxia-induced increase of contractility, the response of the goldfish heart to hypoxia was evaluated before and after treatment with the PI3-K inhibitor, Wortmannin (10^−9^ M). As indicated in Figure 2a, Wortmannin abolished the increase of SV and SW, suggesting a mechanism that, via a PI3-K-dependent pathway, induces the activation of the NOS/NO system. Consistent with this, Western Blotting analysis revealed, in goldfish hearts perfused under hypoxia, a significant increase of the phosphorylated form of the NOS-controlling protein Akt (pAkt). After treatment with Wortmannin, pAkt values returned to levels comparable to those detected under normoxia (Figure 2b).

### 3.4. NO Intracellular Signals

#### 3.4.1. Role of cGMP

**Figure 3:**
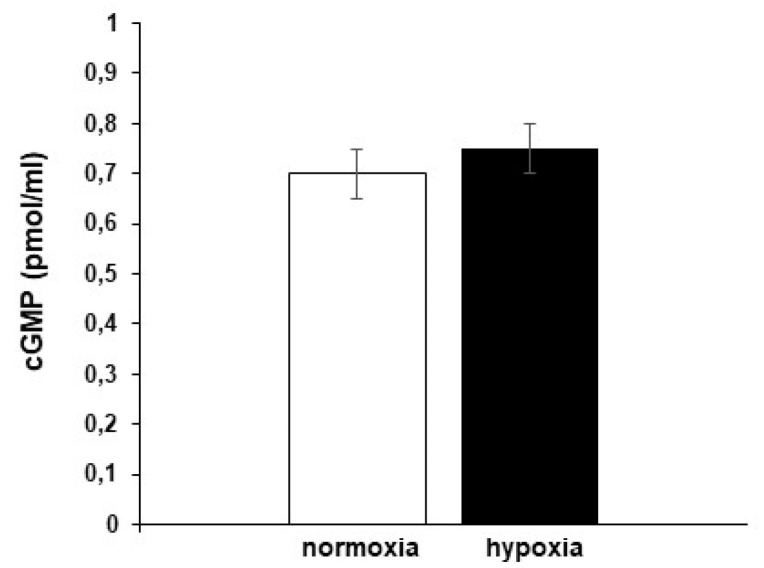
cGMP levels in goldfish cardiac extracts under normoxia and hypoxia. Data were expressed as means ± s.e.m. of absolute values from individual experiments (*n* = 3); statistics were assessed by two-tailed unpaired *t*-test (*p >* 0.05).

NO may affect cardiac performance via the activation of cGMP-dependent pathways [26,39,40,41,42,43]. To assess if, in the hypoxic goldfish heart, the time-dependent increase of contractility involves the generation of cGMP, cGMP levels were measured in homogenates of hearts perfused under either normoxia or hypoxia. The results showed no differences between the two conditions (Figure 3), thus excluding the involvement of this second messenger in the mechanisms used by NO to modulate the response of the goldfish heart to hypoxia.

#### 3.4.2. Analysis of s-nitrosylated Proteins

S-nitrosylation, the covalent modification of protein cysteine thiols by a NO group to generate s-nitrosothiols (SNO), represents a cGMP-independent mechanism modulating many physiological pathways [23,44].

**Figure 4:**
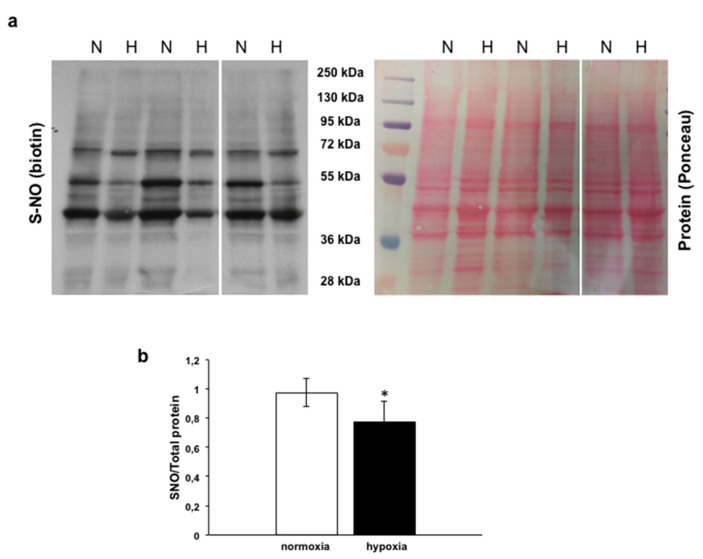
Biotin Switch assay of s-nitrosylated proteins. (**a**) Blots and corresponding Ponceau staining of s-nitrosylated proteins in homogenates of goldfish hearts perfused under either normoxia (N) or hypoxia (H). (**b**) Densitometric analysis of normalized S-NO (biotin)/Ponceau signal. Data were expressed as means ± s.e.m. of absolute values from individual experiments (*n* = 3); statistics were assessed by two-tailed unpaired *t*-test (* *p* < 0.05).

To assess, in the goldfish heart, the pattern of s-nitrosylated proteins, the Biotin Switch assay was used on homogenates of control hearts and of hearts exposed to hypoxia. With respect to the normoxic counterpart, cardiac tissues exposed to hypoxia showed a significant reduction of S-nitrosylation of a broad range of proteins (Figure 4a,b).

#### 3.4.3. Role of SERCA2a Pumps

**Figure 5:**
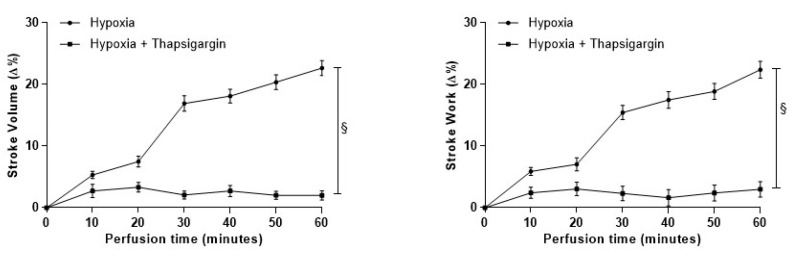
Effects of Thapsigargin in hypoxia-exposed goldfish hearts. Time-course curves for the Stroke Volume (SV) and Stroke Work (SW) of the isolated and perfused goldfish heart before and after treatment with Thapsigargin (10^−7^ M). Data are expressed as mean values ± s.e.m. of 4 experiments. Statistics were assessed by two-tailed unpaired *t*-test (§ *p* < 0.05; hypoxia vs. hypoxia plus Thapsigargin).

Evidence in mammals designated NO as a key modulator of Ca^2+^ cycling, influencing Ca^2+^ channels and SERCA2a pumps [45,46,47]. In fish, a regulatory role of NO on cardiac calcium reuptake by SERCA2a emerged in the eel (*Anguilla anguilla*) [23]. In the goldfish heart, the role of SERCA2a pumps in the response to hypoxia was evaluated by exposing isolated heart preparations to hypoxia in the presence of the specific inhibitor Thapsigargin (10^−7^ M). The treatment significantly reduced the time-course increase of contractility in hearts exposed to hypoxia (Figure 5), indicating that the nitrergic modulation of the goldfish heart, in response to low O_2_, involves a NO-dependent modulation of the rate of Ca^2+^ re-uptake by SERCA2a. In the normoxic goldfish heart, under basal conditions, Thapsigargin (10^−7^ M) per se did not significantly modify basal mechanical performance [36,38].

### 3.5. Nox2 Expression

**Figure 6:**
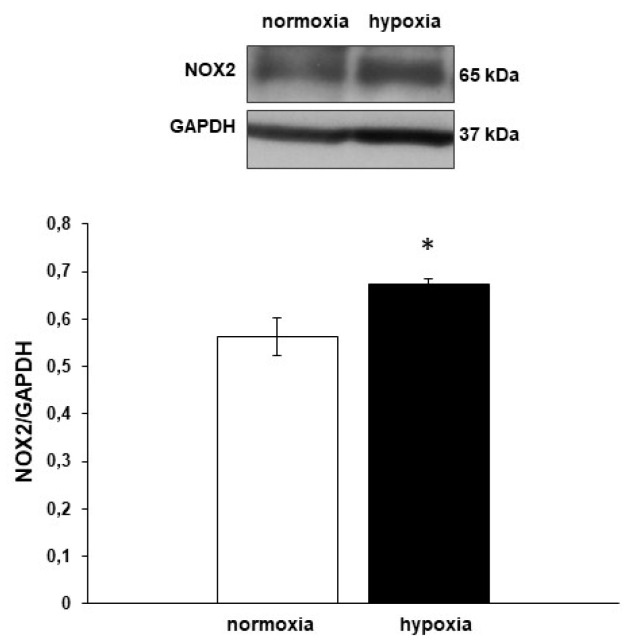
Nox2 (gp91phox) expression in hypoxia-exposed goldfish hearts. Representative Western Blotting and densitometric analysis of Nox2 (gp91phox) expression in extracts of goldfish hearts perfused under either normoxia or hypoxia. Data were expressed as means ± s.e.m. of absolute values from individual experiments (*n* = 3); statistics were assessed by two-tailed unpaired *t*-test (* *p* < 0.05).

NADPH oxidase is an important cellular source of O_2_^-^. In the heart, it is involved in many physiological and pathological processes, including hypoxic adaptation [48]. To investigate whether, in the goldfish heart, hypoxia can influence NADPH oxidase activity, the expression levels of Nox2, the catalytic subunit of the enzyme, were investigated by Western Blotting. As shown in Figure 6, an immunoreactive band corresponding to the predicted molecular weight of Nox2 was detected in homogenates of hearts perfused under either normoxic or hypoxic conditions. In particular, the resulting Nox2 expression was significantly increased in goldfish hearts exposed to hypoxia.

## 4. Discussion

By using the goldfish as a gold standard of hypoxia tolerance, we explored whether the NOS/NO system and the downstream-activated signals provide advantage to the heart under low O_2_. To the best of our knowledge, our data are the first to show that NO sustains the intense contractility of the hypoxic goldfish heart via a mechanism which is independent of cGMP, and involves a PI3-K/Akt-mediated activation of NOS-dependent NO production and SERCA2a pumps. The denitrosylation and/or putative nitration of intracellular targets have also been evaluated as related mechanisms that contribute to the high resistance of the goldfish heart to hypoxia.

### 4.1. PI3-K/Akt/NOS/NO Pathway Activation

The remarkable ability of the goldfish heart to enhance its basal performance when exposed to a hypoxic milieu has been largely documented by studies from our laboratory [7,8,12]. These studies reported that in *C. auratus*, exposure to hypoxia is accompanied by an increased expression of cardiac HIF-1α (hypoxia-inducible factor 1α) and NOS. This expanded to this teleost the protective role elicited by NO on the hypoxic myocardium [12], already proposed in mammalian and non-mammalian vertebrates (see for references [49,50]). In agreement with these results, we now observed a significant reduction of the hypoxia-dependent increase of contractility in hearts perfused in the presence of the NO scavenger PTIO or the NOS inhibitor L-NMMA, which represents the physiological evidence of the NO need in the hypoxic goldfish heart. This was supported by the hypoxia-induced activation of the PI3-K/Akt pathway, a well-known player in the NOS-dependent NO production (see for example [51,52,53]). The involvement of this pathway was shown by the significant reduction of contractility induced by the PI3-K inhibitor Wortmannin, and by the increased level of pAkt in hearts perfused under hypoxia. In line with this, extracts from hypoxic hearts treated with Wortmannin showed pAkt levels similar to those detected under normoxia. In mammals, Akt represents not only the effector of the PI3-K-mediated NOS activation pathway, but also a cardioprotective factor, able to regulate a variety of cell functions under hypoxia [54]. In the ischemic mammalian heart, Akt promotes the utilization of glucose, instead of free fatty acids, and adequate myocardial oxygen consumption [54]. Moreover, in response to hypoxia, the adenoviral gene transfer of activated Akt protects cardiomyocytes from apoptosis [55]. Akt may also improve the contractile function of the myocardium by increasing SERCA2a levels, or by enhancing its activity through the inhibition of phospholamban (PLN) via its phosphorylation [54]. Studies in non-mammalian vertebrates show that Akt plays a role in the cardiac response to environmental, physical and chemical stimuli (eel: [23]; lungfish: [21]; frog: [56,57,58]). In addition, in the hypoxic goldfish heart, Akt is proposed to mediate the effects elicited by Selenoprotein T-derived peptide [38], a cardioprotective factor that in mammals reduces ischemia–reperfusion injury [59]. In line with these observations, our results strongly support the possibility that Akt, in concert with other cardioprotective factors, may contribute to the hypoxia resistance of the goldfish heart.

### 4.2. NO Downstream Effectors

**Figure 7:**
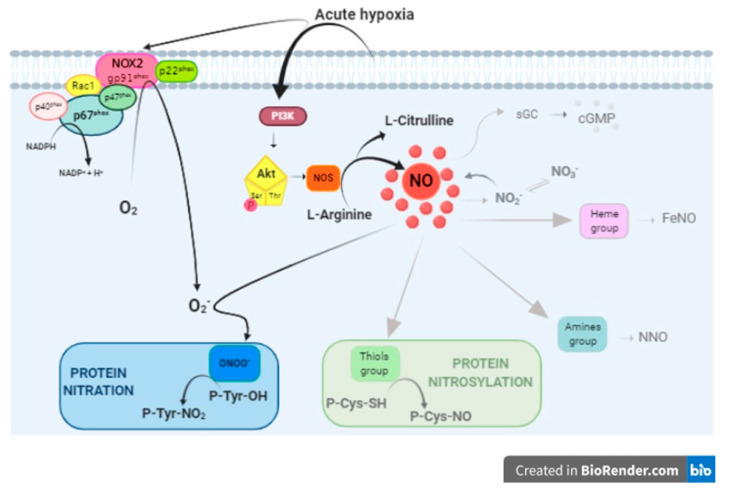
Simplified overview of NO-mediated intracellular pathways. NO may activate the soluble guanylyl cyclase (sGC) to produce cGMP, or interact with heme, amines or thiols to produce iron-nitrosyl (FeNO), s-nitroso (SNO) and n-nitroso (NNO) compounds. On the other hand, NO can react with superoxide radicals (O_2_^−^) to generate peroxynitrite (ONOO^−^). Our results suggest that under acute hypoxia, the activation of the PI3-K/Akt pathway and the Nox2 enzyme may cause the simultaneous generation of NO and O_2_^−^, respectively, thus contributing to protein nitration (black arrows).

A complex chemistry and target factors are involved in the NO-mediated intracellular effects. Under hypoxia, this picture is further complicated by the presence of reactive oxygen species and their connection with NO-related products (Figure 7).

With the aim of disentangling the mechanisms activated in the hypoxic goldfish heart downstream NO production, we analyzed the involvement of cGMP, the classic NO mediator. In the working goldfish heart, the NO-induced cGMP generation significantly affects mechanical performance, by tonically decreasing SV under basal conditions [12]. However, data obtained in the present study excluded the involvement of cGMP in the mechanisms responsible for the time-dependent increase of myocardial contractility experienced by goldfish under low O_2_, as indicated by the comparable levels of cGMP detected by the ELISA test in homogenates of hearts perfused under either normoxia or hypoxia.

Accumulating evidences indicate that major NO-mediated non-cGMP signals are related to the covalent attachment of NO to cysteine (Cys) residues (s-nitrosylation) [60]. In fish, the pattern of s-nitrosylated proteins was studied by our research group through Biotin Switch assays in eel (*A. anguilla*) cardiac tissues, in response to both nitrite [61] and preload [23] stimulation, showing an increase of protein s-nitrosylation. By using the same experimental approach, we have now found, in goldfish hearts exposed to hypoxia, a significantly reduced amount of s-nitrosylated proteins with respect to their normoxic counterpart. About 3000 proteins have been identified as targets of s-nitrosylation [62], indicating the importance of controlling this mechanism for a proper cardiac function. Accordingly, dysregulated protein s-nitrosylation has been correlated with several heart, muscle and lung diseases, as well as cancer and neurodegenerative disorders [63,64]. By using transgenic mice with cardiomyocytes overexpressing the denitrosylating enzyme s-nitrosylated glutathione reductase (GSNOR), Sips et al. [65] proposed protein denitrosylation as a protective mechanism against myocardial dysfunction under stress. Works are in progress in our laboratory to identify proteins encountering denitrosylation in the hypoxic goldfish heart, and their related functional significance. While waiting for more detailed information, in agreement with the mammalian data, the present results propose denitrosylation as a mechanism that in fish is activated under conditions of hypoxic stress to sustain cardioprotective programs.

It is known that, in the presence of excessive reactive oxygen species, NO forms RNS. In particular, the fast reaction of NO with superoxide (O_2_^−^) leads to peroxynitrite (ONOO^−^) production (Figure 7) [66]. Peroxynitrite-mediated protein modifications include tyrosine nitration, the substitution of a hydrogen by a nitro group in the position 3 of the phenolic ring, generating 3-NT. This may alter protein catalysis, protein–protein interaction, and tyrosine kinase signaling [67]. However, rather than inducing protein damage, nitration is proposed as a control mechanism of redox homeostasis in normally functioning cardiac muscle [68]. Interestingly, by Western Blotting analysis, we observed, in the high-performing hypoxic goldfish heart, an increase of Nox2 expression, indicative of an increased NADPH oxidase activity. This evidence, which agrees with the increased (but not detrimental) levels of 3-NT we previously observed in goldfish hearts exposed to hypoxia [38], supports the possibility that nitration contributes to the high resistance of the goldfish heart to conditions of reduced oxygen. Tyrosine nitration is a highly selective process, since neither all proteins nor tyrosine residues of a protein are nitrated. It has been reported that in whole tissue/cells, only 1–5 out of 10,000 tyrosine residues may be nitrated [19]. However, several proteins show numerous nitrated tyrosine residues with consequent structural and functional changes [19,69,70]. In this context, our data are of interest since they provide a conceptual basis to explore the apparent contradiction between the established benefits of NO supplementation under hypoxia, and the general concept of RNS, and related downstream-activated cascades, as deleterious for the cells.

An important intracellular target of NO is represented by SERCA (see for example [71,72,73,74]), the integral membrane protein controlling Ca^2+^ homeostasis through its active transport across the sarcoplasmic reticulum. In cardiac muscle, SERCA2a is the predominant isoform. By ensuring sufficient Ca^2+^ load in the sarcoplasmic reticulum, it modulates muscle relaxation as well as contraction [75,76,77]. SERCA2a is regulated by PLN, which, when de-phosphorylated, is bound to the pump, and this decreases the affinity for Ca^2+^ [78,79]. When phosphorylated, PLN dissociates from SERCA2a, thus restoring its affinity for Ca^2+^ [79]. SERCA2a pumps are susceptible to oxidative and nitrosative modifications, as they contain vulnerable cysteine and tyrosine residues (see, for reference, [68,80]). The nitrotyrosine modification of SERCA2a has been observed in several pathophysiological conditions [73,81], and nitrated SERCA2a has been recently considered as a cardiac marker of nitrative stress [68]. It has been proposed that the close proximity of SERCA2a and mitochondria exposes the pump to reactive oxygen/nitrogen species, which are derived from superoxide generated as a by-product of mitochondrial oxidative phosphorylation [82], also providing a way to regulate energy metabolism under stress conditions [68].

The nitration of specific proteins, including SERCA2a, was not specifically assessed by the present study. However, the abolition of the hypoxia-dependent increase of the cardiac performance induced by SERCA2a-specific inhibition by Thapsigargin clearly suggests a mechanism which involves SERCA2a-controlled muscle relaxation. Interestingly, in fish, the amino acid sequence of SERCA2a (zebrafish: NP_957259.1) includes tyrosine residues (i.e., 294-295 and 753), which in mammals are recognized as potential sites for nitration. This opens another suggestive route for investigations.

## 5. Conclusions

In conclusion, the proposed study revealed novel aspects of the still-unresolved mechanisms that sustain the elevated hypoxic tolerance of the goldfish heart. We showed the involvement of a PI3-K/Akt/NOS/NO cascade that escapes the classic cGMP generation, but is paralleled by the SERCA2a pumps’ activation and increased expression of Nox2. Remarkably, for the first time, protein s-denitrosylation was found to be associated with the exposure of the goldfish heart to low O_2_. A dynamic balance between protein nitrosylation and denitrosylation is critical for a proper myocardial functioning, also in response to stress [83]. Further studies will clarify the significance of denitrosylation in the goldfish heart challenged by hypoxia, for example by identifying the specific proteins that undergo denitrosylation. Another point to be resolved will be the apparent contradiction between NO generation and denitrosylation. As we suggested, NO-dependent protein nitration may represent a concurrent phenomenon that enhances the spectrum of opportunities for NO to protect the stressed goldfish heart.

## 6. Limitation of the Study

The available data and our results do not allow us to identify the specific NOS isoform/s involved in NO production in the hypoxia-exposed goldfish heart. Mammalian cardiomyocytes express both nNOS [84] and eNOS [85], whose differential biological functions are tightly related to intracellular compartmentation, differences in their stimulation, and specific recruitment of distinct downstream transduction pathways (see [86] for references). In teleost fish, as well as in agnathans and chondrichthyans, while physio-pharmacological approaches and NADPH-diaphorase and immunolocalization studies have documented the presence of an “eNOS-like” activity in the heart of several species [28,86,87,88], a gene for a canonical eNOS has been yet not identified (see for reference [86,89]). It has been proposed that in teleost, a nNOS isoform showing an endothelial-like consensus may cover some functional features of the eNOS isoform identity. However, this aspect remains a hindrance to completely understanding the role of the NOS/NO system and related nitrosative signals in the hypoxic goldfish heart. The authors leave such efforts to targeted studies.

## Figures and Tables

**Table 1:** Baseline cardiac parameters of the isolated goldfish (*Carassius auratus*) heart, perfused under either normoxia or hypoxia.

Cardiac Parameters	CO (mL min^−1^ kg^−1^)	SV (mL kg^−1^)	HR (Beats min^−1^)	SW (mJ g^−1^)	Preload (kPa)	Afterload (kPa)
Normoxia	13.531 ± 0.379	0.186 ± 0.016	76.333 ± 5.459	0.235 ± 0.020	0.073 ± 0.002	1.413 ± 0.018
Hypoxia	13.777 ± 0.479	0.194 ± 0.017	76.769 ± 6.299	0.288 ± 0.032	0.064 ± 0.006	1.434 ± 0.031

Values are means ± s.e.m. of 9 (normoxia) and 13 (hypoxia) experiments. CO: Cardiac Output; SV: Stroke Volume; HR: Heart rate; SW: Stroke Work.
